# Appdaptivity: An Internet of Things Device-Decoupled System for Portable Applications in Changing Contexts

**DOI:** 10.3390/s18051345

**Published:** 2018-04-26

**Authors:** Cristian Martín, Jeroen Hoebeke, Jen Rossey, Manuel Díaz, Bartolomé Rubio, Floris Van den Abeele

**Affiliations:** 1 Department of Languages and Computer Science, University of Málaga, Boulevar Louis Pasteur 35, 29071 Málaga, Spain; mdr@lcc.uma.es (M.D.); tolo@lcc.uma.es (B.R.); 2 IDLab, Department of Information Technology (INTEC), Ghent University-IMEC, Technologiepark-Zwijnaarde 15, 9052 Ghent, Belgium; jeroen.hoebeke@intec.ugent.be (J.H.); jen.rossey@intec.ugent.be (J.R.); floris.vandenabeele@intec.ugent.be (F.V.d.A.)

**Keywords:** IoT, CoAP, application portability, 6LoWPAN, data flow programming

## Abstract

Currently, applications in the Internet of Things (IoT) are tightly coupled to the underlying physical devices. As a consequence, upon adding a device, device replacement or user’s relocation to a different physical space, application developers have to re-perform installation and configuration processes to reconfigure applications, which bears costs in time and knowledge of low-level details. In the emerging IoT field, this issue is even more challenging due to its current unpredictable growth in term of applications and connected devices. In addition, IoT applications can be personalised to each end user and can be present in different environments. As a result, IoT scenarios are very changeable, presenting a challenge for IoT applications. In this paper we present Appdaptivity, a system that enables the development of portable device-decoupled applications that can be adapted to changing contexts. Through Appdaptivity, application developers can intuitively create portable and personalised applications, disengaging from the underlying physical infrastructure. Results confirms a good scalability of the system in terms of connected users and components involved.

## 1. Introduction

We are witness to an unprecedented and rapid evolution in electronics, wireless communication and miniaturisation technologies. This has contributed significantly to reducing the production costs of electronic products and embedded devices, thereby increasing the number of them in the Internet era. Nowadays, it is possible to acquire a personal computer from 9 dollars [1], something unachievable a few years ago. These devices are usually devices connected to the Internet with capabilities to sense and actuate over the physical world, paradigm also known as the Internet of Things (IoT) [2]. According to various research reports [3], the number of connected devices is predicted to reach anywhere from 20 to 50 billion by 2020. This huge growth has to be necessarily supported by standard mechanisms and protocols [4], otherwise it will result in multiple vertical silos for multiple applications, complicating the IoT adoptions.

Many IoT devices are designed to be working for long periods and are battery-powered since they can be deployed in environments where there may not be electricity (e.g., environmental monitoring). To reduce the power consumption and production costs, these devices have limitations in term of storage, processing and networking. Therefore, applying current standard ways to intercommunicate systems and exchange information such as web services is a non-viable task in most cases. Moreover, one of the main challenges in the moment of designing an IoT application is the vendor lock-in issues derived of using multiple platforms without a common backbone standard. The Internet Engineering Task Force (IETF) has taken into account the aforementioned problems and has contributed with many standards intended to reduce the gap between resource-constrained devices and powerful ones and connect these devices directly to the Internet. One of its results is the IPv6 over Low power Wireless Personal Area Networks (6LoWPAN) [5], which enables the transmission of IPv6 packets on top of IEEE 802.15.4 networks. IPv6 packet sizes are much larger than those in IEEE 802.15.4 networks, and mechanisms for compression/decompression are needed to enable IPv6 use in constrained networks. Routing is also a challenge in constrained networks since they have special features such as limited radio range, large number of nodes and limited resources. A IPv6 Routing Protocol for Low-Power and Lossy Networks (RPL) [6] was also released by the IETF to solve the routing issues in constrained networks. The IETF also created the Constrained RESTFul Environments (CoRE) working group to bring REST web services to constrained devices, releasing the Constrained Application Protocol (CoAP) [7].

Despite the improvements of the latest protocols for the IoT, IoT applications are still designed to be coupled to final devices, which means that applications do not adapt to the continuous changes in the underlying infrastructure. In the case of inclusion of new devices to these applications or even their disconnection due to battery consumption, it is still necessary to reconfigure these applications and devices to adapt to these changes and to be part of the system. In the IoT where the number of devices grows in a unprecedented way and devices are very volatile, these limitations drag out its expansion. For instance, in environmental monitoring, device-decoupled applications could display over-the-fly measurements from sensors deployed based on the users’ needs. Moreover, IoT applications are personalised, i.e., the underlying IoT infrastructure can be designed to work with certain end users. This greatly increases the complexity in the application development. For instance, imagine a personalised application for more than one hundred end users. The development for all the personalised logic can represent a higher development effort than the whole architecture itself. Last but not least, end users can be part of different IoT applications in separate scenarios. In this case, it would be desirable to have a control over these applications with the minimum configuration and using a common interface. Appdaptivity is intended to solve these challenges, providing a framework for the development of IoT applications that are:

- portable
- personalised
- adapted to changing contexts
- created intuitively

This work is based upon well-established standards so as to avoid a new vertical silo in the IoT. This approach is intended to offer a novel framework for developing portable, personalised and adapted to changing contexts CoAP-based applications with a minimal configuration. The IoT comprises other application protocols such as MQTT, DDS and AMQP [4]. Appdaptivity is a component-based system, thereby these protocols can also be part of the underlying IoT infrastructure with new integration components. It should be noted that this work uses an efficient access of sensors and actuators through CoAP functionalities such as the observe operation and the group management, which could not be found in other IoT protocols. However, the integration of IoT protocols would enable the interaction with non-CoAP systems, e.g., an actuation with an external system. The developed applications offer a set of behaviours than can be updated or created based on the device changes during the system execution, adapting applications to the environment changes. The application development is done with a visual tool where users can intuitively create flows, following action sequences. It has been shown how visual environments provide a better user experience, alongside a higher perceived success and a reduced perceived workload [8], leading to better results [9]. Last but not least, the security has been analysed over the life cycle of the system and a secure solution using standard protocols that covers all the components involved has been proposed.

The rest of the paper is organised as follows. An introduction of CoAP is given in Section 2. In Section 4 we discuss related work on device-decoupled applications. The problem statements and research goals of this work are highlighted in Section 3. Section 5 presents an overall overview of the Appdaptivity architecture and its components. Then, the implementation of Appdaptivity is provided in Section 6. The Appdaptivity evaluation has been done in Section 7. Section 8 discusses the differences between CoAP, the CoAP++ framework and Appdaptivity. Lastly, Section 9 presents our conclusions and outlines future work.

## 2. Constrained Application Protocol (CoAP)

Heterogeneity, limitations and vendor lock-in issues have been present for a while in resource-constrained devices. As is mentioned above, the IETF has contributed with many standards to connect these devices to the Internet and reduce the gap between them and powerful ones. Among these standards it should be pointed out 6LoWPAN and RPL. However, the advances in the media layers are not enough, since the heterogeneity still climbs to the upper layers. Taking note of the expansion of the World Wide Web (WWW) and its standard interconnection mechanisms (web services), the IETF designed, through its CoRE working group, the Constrained Application Protocol.

**Figure 1:**
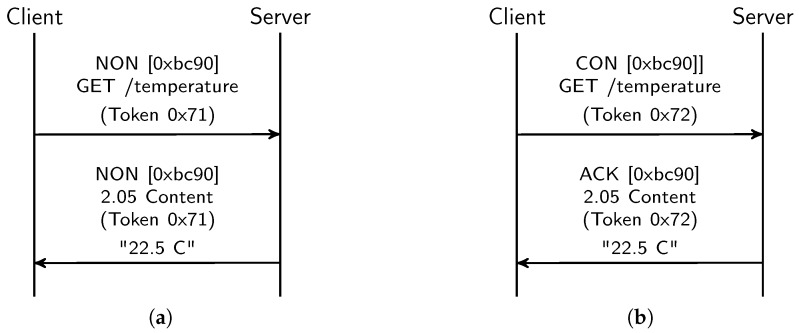
CoAP messages. (**a**) Non-confirmable CoAP GET request; (**b**) Request with piggybacked response.

CoAP provides a RESTful architecture similar to current web services, nevertheless it has been adapted to resource-constrained devices. The most notable feature with respect to current RESTful web services is the abolition of the high-consumption protocols involved, such as HTTP and TCP. Handshaking, packet reordering and header characteristics of these protocols suppose a high barrier to devices with limited capabilities and presence in constrained networks. CoAP has adopted UDP as the transport layer, thereby reducing the overhead and requirements of TCP communications. The adoption of UDP brings IP multicast which can improve the control and reduce the number of communications in constrained networks. Due its UDP nature, CoAP provides two types of communications: confirmable and non-confirmable messages. Therefore, CoAP offers the strengths of confirmable messages in TCP while simutaneously providing lightweight communications for environments where confirmations are not necessary (e.g., stream data monitoring). To reduce the number of communications, confirmation responses can also be piggybacked. With a client-sever model, Figure 1 shows two examples of CoAP communications. Concretely, a client sends a GET request with a non-confirmable message to a CoAP server, and the server returns the temperature status (Figure 1a); and the same communication with a confirmable message and a piggybacked response (Figure 1b).

**Figure 2:**
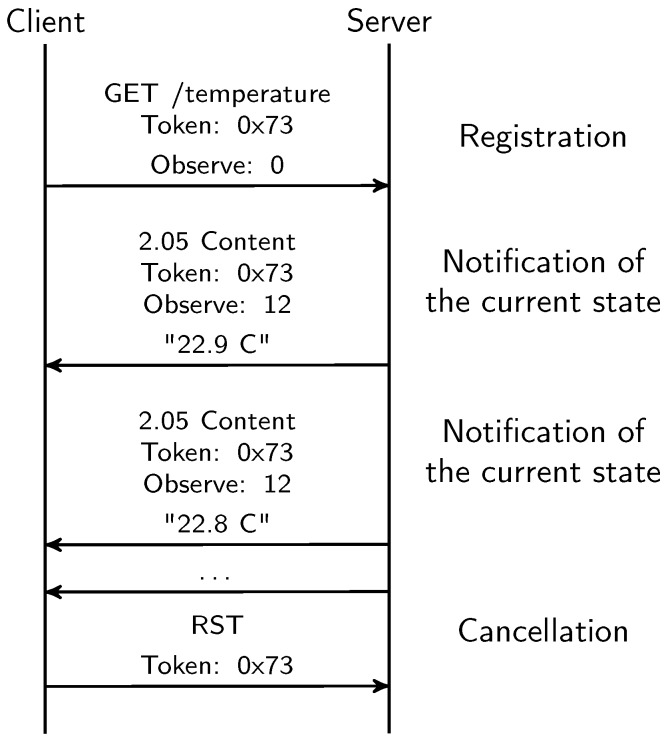
Observing a resource in CoAP.

Like RESTful web services, CoAP offers operations and objects through resources, which can be accessed with HTTP-style methods (GET, PUT, POST and DELETE). Accessing sensors and actuators can be defined in resources, thereby enabling an HTTP-style access to them. For instance, in Figure 1 a CoAP server contains a temperature sensor resource which is accessed through the ‘/temperature’ URI by CoAP clients. In monitoring systems, it is important to have an up-to-date representation of the interested resources. However, continuous resource polling can drastically affect the devices’ performance since communication is one of the most power-consumping tasks on these devices. CoAP supports proxying with caching capabilities, which can reduce the number of communications with final devices. Consider an HTTP-CoAP proxy which translates HTTP requests into CoAP ones. In the latter scenario, HTTP clients can access CoAP resources in the same way as the WWW, enabling the known paradigm of the WoT. However, it does not make an efficient use of these resources. IoT devices have still limitations that they have to address, like sleeping cycles which complicate access by clients. Furthermore, most monitoring systems are not interested in receiving monitoring data, but they are interested in data changes. An extension of CoAP, known as resource observation [10] introduces a mechanism to optimise the interactions with CoAP devices. Resource observation enables a new asynchronous communication model in CoAP where clients can subscribe to resources and receive asynchronous notifications. The observe operation can be configured with a max age option to indicate an age up to resource statuses are acceptable. Resource notifications are also sent when its status changes. Therefore, the resource observation avoids continuous client polling in resources and final devices can send data when they are available combining it with other tasks like the sleeping mode. On the other hand, clients can receive asynchronous data updates without polling resources. An example of a observe communication is given in Figure 2. The communication starts with a GET request to the resource of interest by a CoAP client, a temperature sensor resource in this case, with the observe option established. Once the CoAP server has processed the request, it saves the client in the list of observers and sends observe updates until the client sends an RST message. Resource observation can also be optimized with conditional observations [11] where clients can establish a configurable criteria to receive resource notifications.

## 3. Problem Statement and Research Goals.

The IoT is continuously expanding in a unprecedented way, changing the way we interact with each other and with the environment. Many companies have adopted this epoch-defining revolution to improve their business processes and manufacturing chains with the capabilities offered by the IoT. This revolution has also reached many other environments such as housing and buildings, healthcare, cities and farming, to name but a few [2,12,13]. However, most have resulted in vertical silo applications with established and predefined physical IoT devices. This makes the inclusion of new components at run-time difficult and goes against one of the IoT philosophies of having ready-to-use devices with minimal configuration. Although some systems provide discovery and joining mechanisms, forming ready-to-use devices, applications are still dependent on the physical devices and reconfiguration is usually required upon the inclusion or disruption of devices. Application logic should be abstracted from the underlying physical infrastructure adapted to changing contexts, automatically adapting the application logic to the underlying infrastructure changes.

IoT devices are becoming more personalised. The advances in the IoT have contributed to the release of a wide range of devices for multiple areas. These devices apart from being shared between the community (e.g., a city CO2 sensor), also belong to people (e.g., a door locker). The development of IoT applications should take this into account as part of their design, but without deviating from their main goal: the application logic. Otherwise, the development for highly personalised applications would represent a high effort.

In large IoT deployments, it may be a challenge to define the application logic. If the IoT deployment comprises multiple infrastructures, the application logic is normally installed and configured in each one of those scenarios, again making the expansion of the IoT difficult. If instead of having to configure each environment, these environments could be dynamically configured and be part of the IoT applications, the IoT applications would not have to address the challenges of having multiple infrastructures. This would enable the portability of applications for heterogeneous environments.

The application development itself can also represent an obstacle for the development of IoT applications. The IoT has been taken up by multiple kinds of users, both with and without expertise. This means that the spread of the IoT would be limited if the application development required the use of complex abstractions. To reach a wide variety of end users, these applications should be intuitively created, i.e., the development should be easy to do and not represent an obstacle for end users.

Finally, as it is known that IoT devices are subject to a set of limitations. The work should focus on devices with low capabilities, like Class 1 devices (≈10 KB RAM and ≈100 KB ROM) that can be considerably reduced with networking stack libraries and the application logic. Therefore, the solution should affect these resource-constrained devices as little as possible, otherwise it cannot be applied.

Our research aims to answer the following question based on the problems identified in the development of IoT applications:

(i) How can we intuitively define portable, adaptable, personalised and device-decoupled IoT applications?

## 4. Related Work

Programming wireless sensor networks has garnered a lot of attention due to its complexity, since it requires knowledge in multiple fields, such as distributed computing, embedded devices and wireless networks. Multiple abstractions have been released to reduce this gap, such as service-oriented, data-driven and group-based programming. The idea behind device-decoupled applications can be related to the macro-programming paradigm in wireless sensor networks [14,15]. Macro-programming allows sensor networks to be programmed as a whole, rather than programming low-level software for individual nodes. This was a great advance in the development of sensor networks, and greatly reduced the programming efforts in large sensor networks. However, most approaches like EcoCast [16] do not focus on open standards, making their implantation in the continuous-growing IoT difficult. Our approach in Appdaptivity follows a similar philosophy to macro-programming, adapting it to current standards in the field, such as CoAP.

The dependency between devices and applications could be carried to its maximum by directly moving the application logic inside the sensor networks. This approach considerably reduces the latency and the number of packets, since communications are carried out inside the Low power and Lossy Networks (LLNs) and there is no need for communications with external components. In addition, the dependency on other factors such as routing and network reliability in cloud-based applications is reduced. This paradigm is known as in-network processing. Bindings and RESTlets [17] are extensions of CoAP designed by the IDLab research group, which introduce flexible bindings between sensors and actuators and application building blocks respectively. RESTlets configure the inputs, output and some processing tasks just using CoAP on the devices enabling in-network applications which use direct binding between sensors and actuators. T-Res [18] also proposes an extension of CoAP to enable in-network applications. T-Res improves the application logic, which enables scripts for defining custom algorithms to be programmed. However, it requires the use of an embedded Python virtual machine and stores URLs of input sources and destination outputs, which can affect the performance of resource-constrained devices. These solutions optimise the device interactions and reduce the latency in its communications, nevertheless they do not leverage the changes on the environment to adapt the applications.

A Distributed Data Flow (DDF) programming model to develop IoT applications for fog computing is proposed in [19]. In data flow programming, the application logic is expressed through a directed graph of nodes. Nodes define a set of inputs and outputs and an independent function which does not affect to the rest of the nodes. Thus, nodes are highly portable and reusable for the creation of user applications. DDF addresses the interconnection of cloud, edges, IO and compute nodes which comprise fog computing applications. These interconnections enable different interactions such as things-to-things, fog-to-cloud and cloud-to-fog. Thing functionality is provided through specialized nodes by domain experts. Application developers only need to focus on data flow applications. Glue.things [20] presents an IoT mashup resulting from the COMPOSE European project. Glue.things brings three tools for the application development: the device manager, the composer and the deployment manager. Device manager provides a web-based application to connect IoT devices using different protocols (HTTP, STOMP, MQTT and CoAP). Once the IoT device has been connected to the system, it is available for the composer component to create application logic using a web-based data flow model. Lastly, the deployment manager controls and deploys the mashup applications resulting from the composer component. DDF and glue.things have similarities to our work in that they were built on top of Node-RED [21], a browser-based editor that allows visual data flow programming. However, both of them have only focused on the application development instead of providing a full solution with customisable user applications as Appdaptivity does. The most differentiated part of these approaches and others such as Apple Home, Google Home and BACnet, with respect to our work is the application’s adaptability to the underlying physical infrastructure without having to connect these devices and recompile the system. This approach is intended to offer a novel framework for developing CoAP-based applications without reconfiguration. Appdaptivity enables the design of applications that are independent of the underlying physical devices and can be easily adjusted without recompiling. The IoT device has to be manually connected in glue.things and DDF and discovery are not supported, whereas Appdaptivity provides run-time discovery and update services that deploy portable user applications based on the IoT devices available at any given moment.

PatRICIA [22] is a novel programming model for the IoT which decouples physical devices from user applications. PatRICIA introduces two new abstractions: Intent and IntentScope, which represent desired tasks to be performed in a physical environment and logical group of physical devices respectively. Intents are abstract representations of tasks and are applied on IntentScopes. Device controlling and monitoring are defined by domain experts using tasks through device services. On the other hand, application developers define device-decoupled applications using Intents which are dynamically transformed into tasks based on the information contained. Therefore, application developers define device-decoupled applications which use device-coupled applications written by domain expert users. Our solution differs from PatRICIA in the programming model. Instead of having different roles of development which decouple applications from devices only once, Appdaptivity has just one programming model which enables device-decoupled programming with visual components which leads to better results [8,9]. Appdaptivity is based upon a standard and promising IoT protocol, CoAP, with more than 30 open source implementations for IoT devices [23]. This increases the adoption of Appdaptivity as a application development system in the IoT unlike PatRICIA. Moreover, it has not been demonstrated how portable and personalised applications in PatRICIA could be achieved. Lastly, the programming model for device-decoupled applications in PatRICIA requires a familiarity with Intents programming, which increases the development efforts in comparison to visual component programming.

A macro-programming framework for wireless sensor networks is also proposed in PyoT [24]. PyoT hides the complexity of the network by providing the available resources as a set of Python objects for the application developers. A Web UI (User Interface) has been incorporated into the system where users can carry out basic operations such as sensor monitoring, actuation control and resource listing. Therefore, application logic can be defined in two ways: a shell interface where application developers use the Python Application Programming Interface (API) to interact with the CoAP resources, and the Web UI for basic operations. PyoT shares some similarities with respect to Appdaptivity. On the one hand, PyoT uses CoAP as application protocol and enables automatic discovery of available resources and event actions to be performed when they are detected in an abstract way for end users. On the other hand, PyoT provides a high-level abstraction framework for developing IoT applications which reduces the aforementioned difficulties in programming such devices. Lastly, although more limited, a Web UI enables the application development for non-expert users. As has been pointed out in future work, PyoT does not support 6LoWPAN nor CoAP group abstraction, unlike Appdaptivity, thereby it can decrease both the network and device performance. Finally but not least, the CoAP implementation does not provide support for Datagram Transport Layer Security (DTLS) and the solution could be vulnerable in real IoT deployments such as buildings with access control management.

The importance of the separation between the application logic from device firmware is addressed in [25]. This approach also uses CoAP, providing thin servers—devices as a role of CoAP servers without any application logic. Thin servers are provided with embedded metadata (e.g., geographical information, name, brand) to enable a user-friendly and efficient look-up. The programming model is similar to Web 2.0 mashups, enabling the reuse of deployed services and applications on the cloud. Actinium [26] provides a run-time container for dynamic management of application scripts. The resulting applications are modelled as resources themselves, so that they can be combined with other apps resulting in multi-layer applications, CoAP servers or even external applications. Although these approaches abstract the application logic from final devices and make use of standard protocols, they are still not completely agnostic from end devices (they use device services or resources instead), cannot be portable and are not provided with the run-time updates necessary to keep applications up-to-date with changes in the underlying physical infrastructure.

In addition of CoAP, many other application protocols have been released during the last years. MQTT [27] is probably the IoT application protocol which has acquired an attention similar to CoAP. MQTT is a publish/subscribe protocol designed to interconnect things. The comparison between CoAP and MQTT has been deeply studied in the literature [28,29]. Although most studies conclude that the use of each protocol depends on the working scenario and the bitrate, CoAP incorporates RESTful web services that follow the current trends in the Web. It therefore facilitates the integration of the IoT with the Web (WoT), and its observe operation also enables a publish/subscribe model which can be configurable based on the consumer’s needs. IoTivity [30] is a software framework for creating IoT applications that implements Open Interconnect Consortium (OIC) standards. OIC was developed by leading technology companies including Samsung, Cisco and Intel to define standards for ensuring the interoperability of the IoT. AllJoyn, another framework for the IoT was recently integrated with IoTivity. IoTivity is also based on CoAP and provides a connectivity abstraction for other non-IP protocols such as Bluetooth, Z-Wave and ZigBee. That connectivity abstraction would improve the interconnection of proprietary IoT devices. Multiple services allow the configuration of devices, accessing them and creating groups of devices like Appdaptivity. Nevertheless, the application logic is couple with the physical infrastructure. OM2M [31] is an Eclipse project that provides a platform for developing services discovering the underlying network. This provides a RESTful Service Capability Layer (SCL) which offers an abstract layer for handling REST requests. Applications can be created using REST requests that could complicate the application development and a binding protocol for resource-constrained devices like CoAP are not yet available. Kura [32] is another Eclipse project that provides a platform for building IoT applications in gateways. Kura provides a wide range of APIs (including hardware access with protocols such as I2C, USB and GPIOs), which enables the deployment of multiple applications using OSGi, the dynamic component system for Java systems. Kura focuses on gateway IoT applications, therefore it can be part of the underlying physical infrastructure through CoAP and its configurable services can be used for improving the management of the IoT.

## 5. Appdaptivity: An Internet of Things Device-Decoupled System for Portable Applications in Changing Contexts

### 5.1. Requirements

Requirements establish the guidelines for the solution design and should be kept for the solution’s life cycle. Based on the previous research questions and problem statement, the following requirements have been identified:

- Application development completely agnostic from physical devices. Application developers should focus on the application logic instead of considering the final devices that will be part of the system. Final device inclusion and subscription should adapt the logic of the targeted applications.
- Intuitive application development. The IoT is penetrating the consumer market with a large variety of solutions for multiple areas. A minimal device configuration and an intuitive application development are both key to spreading the solution.
- Portable application development. End users can be part of extremely dissimilar environments such as an entire building, a single smart-home and an embedded device. The solution should be able to be dynamically part of these environments with minimal configuration.
- Personalised applications. In some IoT deployments, users have rights to certain devices and cannot interact with any other (e.g., restricted areas). The system should be able to restrict the access to the underlying infrastructure when it is required.
- Adoption of current standards. The IoT requires the use of open standards, otherwise the solution will be taken into a vertical silo, increasing the IoT heterogeneity.
- Embedded solution to reach resource-constrained devices. IoT devices have serious limitations in term of processing, storage and power. The proposed solution should take into account these limitations and not affect their performance.

### 5.2. Approach and Design

As targeted in the aforementioned requirements, the application development should focus on the application logic instead of designing applications for specific devices. Appdaptivity enables the development of IoT applications independently of the physical infrastructure. Through a discovery process, the underlying physical infrastructure is part of the system and the application logic when it is available. Discovery can also be done in multiples scenarios thanks to the involvement of end users. In this case, discovery is dynamically done as a background process and the underlying physical infrastructure can be discovered in multiple contexts. Application logic only has to be done once and this discovery design enables the portability of these applications to multiple heterogeneous environments. Application portability is one of the key features of Appdaptivity.

An abstraction has been adopted to categorise the underlying physical infrastructure and the context changes for end users: the location. The location refers to a logical abstraction where a physical infrastructure has been deployed, e.g., a room. Location enables the modelling of the real work as it has been conceived. All context changes that have happened in locations will be reflected in the application logic defined. This requires that the physical infrastructure has to support the location on their devices in order to correlate them with logical locations. Enabling the location on constrained devices can incur a loss of performance. Here, previous work has been exploited to force/encourage users to configure the location through a virtual resource [33], which does not impact on the resource-constrained devices’ performance. Personalised applications are developed, exploiting previous work on managing access in resource-constrained environments [34], so that only authorised people can access devices. In the discovery process the underlying infrastructure is discovered which a target user has rights to, and the application logic will be fed with these updates. If a certain logic depends on an situation (e.g., temperature values), the application logic will not be activated until all the corresponding components have been received. Therefore, the application development can be globally done for all the personalised applications, since the application logic will be activated depending on the rights of each end user.

Appdaptivity has adopted a data flow programming model [19] to intuitively define user applications. In data flow programming, users define applications through a directed graph of nodes, modeling a flow of actions from which data should be taken. Nodes have a defined function which does not depend on other nodes, therefore they form highly portable and reusable components for the creation of applications. Application developers just need to concentrate on the application logic. Moreover, Appdaptivity provides a large set of nodes to define user applications that can be created without having to program any lines of code. This mechanism is so non-technical users find it easier to use the system, which in turn facilitates the expansion of the proposed solution. Other frameworks provide some of the capabilities to develop device-decoupled, adapted changing contexts and personalised application. To the best of our knowledge this approach is the first to provide a framework for intuitively creating IoT applications that can be portable, personalised and adapted to changing contexts.

Appdaptivity comprises the Portability Core (PoCo), which is the component responsible for the data flow programming in an accessible and transparent Web UI. The PoCo enables location-based application development where users can define their applications. The resulting applications can be dynamically portable to other environments. An adaption for diverse deployment-scenarios has been taken into account and the PoCo offers a variety of deployments for different use cases. The application logic comprises different behaviours in data flow programming, whereas user applications are a set of clients that receive and send data to the PoCo with the information originating from the system and the required information to activate the flows. Behaviours comprise the application logic of the resulting applications and will always be up to date with the underlying contexts. User applications are typically smartphone applications which display an up-to-date representation of the behaviours of the underlying physical infrastructure provided by the PoCo to end users.

IoT devices are another key part of the architecture, and the most restricted one, since it contains resource-constrained devices. For this reason, Appdaptivity has adopted CoAP as the application protocol to interact with final devices. In the scope of this work, there will be sensors to be monitored and actuators to be interacted with, therefore a lightweight standard like CoAP with accessible resources represents a suitable protocol. All the wireless sensor networks with CoAP support that can be part of Appdaptivity will henceforth be referred to as the CoAP network. The CoAP network provides the sensors and actuators and device information which are accessible using CoAP to create IoT applications. The PoCo is responsible for interacting with the CoAP networks and enables the definition of data flows with the obtained CoAP resources that will form different behaviours in the user applications.

**Figure 3:**
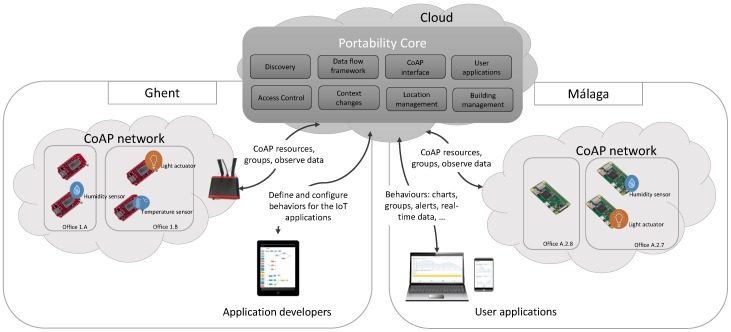
Architecture of the Adaptability.

An overview of the Appdaptivity architecture is presented in Figure 3. In this case, the PoCo adopts a cloud deployment, however it can be deployed in other scenarios as we will see later. In real worldwide IoT deployments, CoAP networks along with their sensors and actuators are deployed in different physical locations, for example different cities, such as Malaga and Ghent. Appdaptivity handles the process of portability of the IoT applications to these environments while at the same time it allows the definition of custom behaviours with a unified and intuitive interface. Discovery is responsible for discovering the CoAP networks in different environments where the portability will be carried out. The behaviours comprise the functionalities that can be defined in the PoCo through its data flow framework and define the actions to be taken with the discovered CoAP networks. Behaviours are executed in the PoCo, however some tasks such as a device interaction can be carried out directly by user applications once the corresponding behaviour has been received. Examples of these behaviours are: the creation of charts with the average value of temperature sensors, alert monitoring based on the sensor data and CoAP groups, for interacting with them with just a unique CoAP request. In the last example, end users could directly interact with the group using CoAP. The CoAP interface in PoCo manages all the CoAP interactions in the system such as a group creation and an observation.

### 5.3. System Deployments

**Figure 4:**
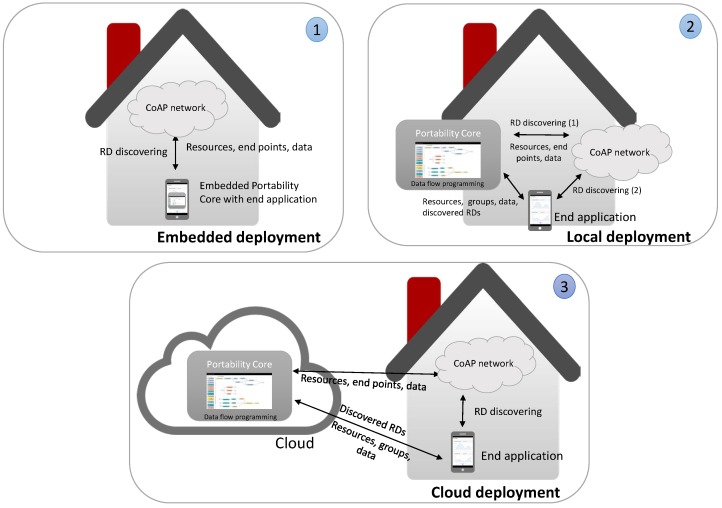
Possible deployment configurations in Appdaptivity.

Depending on the use case and the scope of the application, IoT applications can vary from local use up to worldwide deployments. The portability and access of the resulting applications are different in each use case. The system architecture has been designed to adapt itself to a variety of use cases and enables three ways of deployment (Figure 4):

- cloud (3) : a cloud deployment of the PoCo enables the portability of applications and the access from anywhere with an Internet connection, e.g., access control in an international company. In this case, the discovery of the local CoAP networks by necessity has to be performed by the user applications since the PoCo could be deployed in a different network than CoAP devices and the IP multicast for discovering the CoAP networks has to be performed in the local network. Resource Directory (RD) [35] and CoAP devices will have to be located in the local network and they will have to be IP-accessible in order to enable their discovery and interaction respectively. The RD is an entity launched by the CoRE group which holds information on other servers such as list of resources and their characteristics. RDs are mainly used to indirectly discover nodes when it is not possible or practical due to the given network’s specific characteristics, in this case RDs provide the resources and end points contained in the CoAP network to the PoCo.
- local (2): in this case, user applications and the PoCo have been deployed in different devices. The PoCo will run on a dedicated server, but in the same network as the user applications, e.g., controlling the sensors and actuators of a building on behalf of the employers. The application portability can done with the available CoAP networks in the local network. Discovering of RDs can be done by both user applications and the PoCo.
- embedded (1): user applications and the PoCo have been deployed in the same device. This enables a portable and embedded solution that can be applied to controlled areas, e.g., smart home controlled by the owners. CoAP networks are discovered by the PoCo. In this case, the portability is done in situ with the discovered CoAP networks.

### 5.4. CoAP Network

The CoAP network comprises a set of CoAP servers deployed in known locations that contains a set of resources with the actuators and sensors available for IoT applications. A CoAP server is an IoT device running a CoAP server that collects data and performs actuation in its resources. These CoAP servers have to be previously registered with the RD which enables the observation of the CoAP servers resources and end points by the PoCo. Apart from the sensors and actuators, CoAP servers should define the physical location to enable the user interaction in different locations. CoAP servers could require an extra resource for the location, and this can affect the performance of constrained devices. However, the location can be established through sensor virtualisation [33,36], through which resources are defined in the gateways and it does not affect the performance of the resource constrained CoAP servers.

Once a CoAP server has been started, it can discover the RDs in the network. In the case of an RD discovery, CoAP servers can register its resources in the RD through a POST request as defined in the standard. In the PoCo an observation to the resources and end points of each discovered CoAP network is sent to maintain an up-to-date status of the CoAP networks. Therefore, the system is self-adaptive to network changes and user applications always contain up-to-date behaviours based on the current CoAP networks.

### 5.5. Portability Core (PoCo)

IoT applications are designed using the visual data flow framework provided by PoCo. These applications define the behaviours that will be rendered in the user applications without any configuration by end users, allowing the portability of them to multiple environments. The application developers only need to focus on high-level functionalities, rather than the implementation of low-level details of the underlying physical infrastructure. The service continuity can be a key challenge in applications with large IoT deployments since they can involve multiple heterogeneous networks. In that sense, the PoCo provides the mechanisms to maintain the service continuity in IoT applications independently the underlying physical networks used by the applications. Application can be portable to multiple environments and networks, thus applications are independent of the underlying physical devices and can adapt to the changing context without recompiling, keeping the service continuity of IoT applications in multiple scenarios.

The discovery process used in Appdaptivity follows the OMA LightweightM2M (OMA LWM2M) standard [37], which enables the association of sensors and actuators with an open specification, i.e., resources are identified and organised based on the rules established in the standard. The use of standards allows a unified identification of resources. Nevertheless, apart from the OMA LWM2M standard, other standards like the IPSO Application Framework can also be added and used in Appdaptivity. In case of the adoption of another standard, the specification of the location resource should be configured in Appdaptivity according to the new standard. Moreover, user applications will have to integrate the standard in their UIs in order to know the type of behaviours received and display them accordingly. Moreover, CoAP discovery can be enhanced for supporting semantic matchmaking via inference services and logic languages like the SWoT framework [38].

The work flows that can be configured in Appdaptivity are not only restricted to location-based flows, they can also dispatch the resources belonging to the discovered RDs. Appdaptivity enables two main flows: the location and the building flows. Both flows take as input the resources discovered and the locations of each end points. The location flow is responsible for enabling the behaviours defined in one physical location no matter what RDs have been found; whilst the building flow enables the behaviours defined with all the locations in the RD received, e.g., an entire building. End users can establish their location, or just get their available behaviours in the building based on the last RD received. In order to reduce the number of communications with the RDs and CoAP servers, flows are only activated once end users have established their locations or start the building flow. On the other hand, personalised behaviours are crucial when the number of sensors and actuators increases. This is more important in scenarios where sensors and actuators are restricted, e.g., access control in secured areas.

The Algorithm 1 shows an overall description of the update functionality in Appdaptivity. Once the IoT infrastructure is discovered, or there is any change in the environment, each active location in the location and building flow is checked to see if its corresponding IoT infrastructure has been changed. In the case that any flow has been changed, its behaviours affected will be updated and user applications will receive an up-to-date representation of them. When location or building flow are activated by user applications, they take the current status of the IoT infrastructure with its corresponding CoAP resources and will be up-to-date through this algorithm during its lifecycle.
**Algorithm 1:** Overview of the update algorithm in Appdaptivity
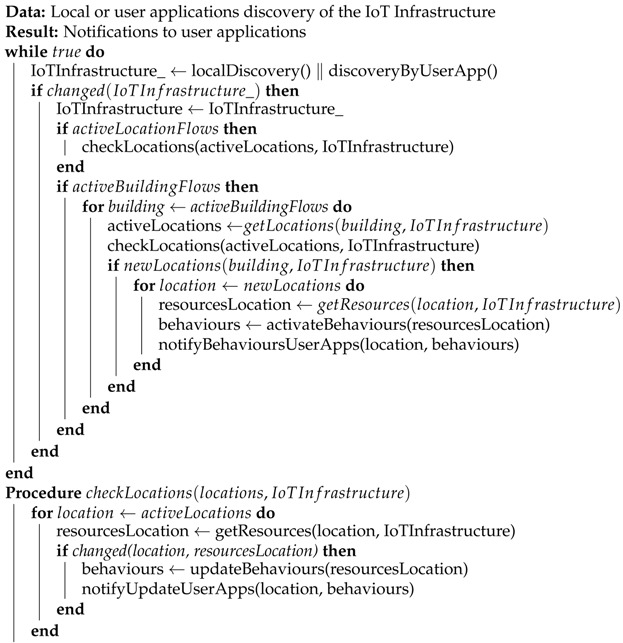


#### 5.5.1. Personalised Behaviours

In personalised behaviours, the communication between the PoCo and the CoAP networks uses the standard DTLS with client certificate authentication, and the communication between user applications and the PoCo uses SSL/TLS. Therefore, the communication channel on both sides can be encrypted, enabling user authentication, and location access control. Security and privacy problems in the IoT, such as applying security patches, physical attacks on the sensor, data leakage, side channel attacks and intrusive sensing are beyond the scope of this paper. To establish a relationship between CoAP networks and physical spaces, the system makes use of the user location to find the nearby infrastructure for each user. The location can be established by end users through the Near Field Communication (NFC) technology. NFC is a short-range identification technology which provides a secure and easy communication and is widely available in multiple devices such as smartphones, thereby it can be used in multiples devices without external hardware requirements. Once a location has been established, the PoCo gets the resources that the user has rights to through a DTLS connection, with client certificate authentication for the CoAP network in the case of a secure configuration. These resources are converted into behaviours for the user applications in the Appdaptivity flows. In a non-secure configuration, the PoCo just returns the available resources of the established location. Tracking user presence in real-time is a hard problem and is also beyond the scope of this paper.

The secure configuration is done through access control lists (ACL) by the physical infrastructure, in this case by the CoAP++ framework; refer to this paper [34] for more information. Encrypted and read-only NFC tags only tell Appdaptivity that a certain user is in a certain location, which can also be done with other identification technologies. Nevertheless, NFC is an identification technology present in many today smartphones, which provides capabilities for security identification. In the case that a user has rights over the resources of this location; the user will receive the associated behaviours to these resources. Therefore, once the location has been established and the user has rights to that location, the authorisation is given indefinitely, i.e., if the user does not establish a new location he/she can move to other physical locations and will have the previously established logic location. Moreover, in the case that users want access to all the locations where he/she has rights in a CoAP network, the building flow give users the opportunity to interact with all their available locations, thus Appdaptivity is not location-strict.

Therefore, in the case that a smartphone application is targeted such as a user application, end users can establish their locations through their smartphones and the NFC tags located in each physical space. When the location has been established, user applications automatically obtain the resources belonging to the location in the terms defined in the behaviours in PoCo, e.g., list of values in real-time, charts, and so on. As discussed, this also requires a configured location resource in each CoAP server to know each server location, but it can be done without a performance lost with EC-IoT [33].

**Figure 5:**
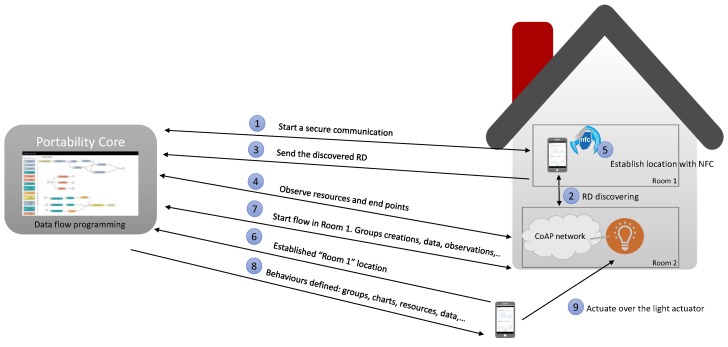
System interaction to actuate with a light actuator in a physical location.

An example of the communication process to interact with a light actuator in a physical location is presented in Figure 5, specifically the system has been deployed in local mode with access control enabled. The communication starts with a connection between an user application and the PoCo (1). Once the connection has been established, the user application starts to discover RDs in the network (2). When a new RD has been discovered, it is sent to the PoCo (3) and an observation to the resources and end points is established (4). From this moment on, end users can establish their location with the NFC tags of each physical Personalised Behavioural space. In the interaction process, the location ‘Room 1’ has been established (5) and sent to the PoCo (6). From this moment, the PoCo starts the location flow in the established location (7) and the user application starts to receive the behaviours defined such as charts, groups and lists of resources allowed (8). Lastly, the end user decides to interact with a light actuator received (9) and a CoAP PUT request is sent directly to the CoAP server with the value selected by the user on the smartphone application.

#### 5.5.2. PoCo Components

The available nodes in the PoCo for data flow programming with which users can define application behaviours are known as PoCo components. PoCo components have a set of inputs and outputs and a given behaviour, e.g., a URI filter component which filters the list of resources received based on the URI information provided in the component. There are some components that need a certain input, as for example, a group component waits for a set of resources to create a new group of these resources. Others can receive inputs from multiple components, such as the component in charge of sending all generated messages to the user applications. Components can be connected with each other in the correct way, forming work flows. Depending on their connections and the components involved, a large number of flows can be generated only with a few components. Therefore, with the same components or a subset of them and different component’ connections, multiple behaviours can be obtained. To avoid functionality interruptions, there are necessary components and connections that are critical to the system (e.g., the discovering process) but there are also many optional components, such as filters which can be included in flows allowing different behaviours.

For the creation of a new component, an HTML file, with its style, and a Javascript file, with its functionality (e.g., filtering CoAP resources), only have to be defined; and then incorporating them to the system configuration. From that moment on, the component will be part of the palette enabling its inclusion in the development of IoT applications. Next, the developed components to define the PoCo behaviours are given:

- Filters: A set of components that filter the list of resources received based on the information established in the components or received in the messages. The filter (resource type, location or URI) information can be configured in each component. Filtering follows open standards for device interaction such as the OMA LWM2M standard [37], thereby it enables a common language in the integration with external systems and user applications.
- Groups: Group is a new entity which aims to address several CoAP resources as a group instead of addressing each resource individually. Groups offer a set of operations for the values of their resources and the possibility to observe them. End users can interact with a set of resources through a group-specific CoAP resource created by this component.
- Group operations: As mentioned above, groups enable a set of operations that can be applied on them. Among these operations are included the minimum, maximum, average and real-time list of resources values. This component makes an observation to the group received with the operation selected and returns asynchronously the information received.
- Observations: Resources can also be observed individually without a group association. This component enables the observation in the resources received and return the data obtained asynchronously.
- Alerts: Sometimes end users can be interested in receiving warnings when something changes in the environment, e.g., a door has been opened. This component checks the data received from the observations and the group operations components and sends an alert message to user applications when the data matches the filters established.
- Actuation trigger: The reaction to conditions established in the environment is addressed by this component. Once an alert has been received based on the established conditions, this component triggers the target resources with the value established in the component to be actuated by the Send request component.
- Charts: This component adds extra information to the data received in order to inform user applications that data should be rendered such as a chart. Otherwise, data is shown without a rendered component.
- Function: Although the goal of Appdaptivity is not to program source code except visually, this component enables adding source code for specific tasks. For instance, a data mining algorithm can be added for data analysis.
- CoAP requests: Once resources or groups have been received in the user applications, they can be requested directly without a PoCo interaction. However, some user applications cannot provide a CoAP support to interact directly with resources or groups. In this case they can send a message to the PoCo to make the requests on behalf of the user application. This component makes a request for the resources or groups received and returns back the operation result.
- Access control: Lastly, this component provides access control to create personalised behaviours. Although this component can be mandatory in most uses cases, sometimes the access control is not necessary, e.g., public sensors or collaborative actuators. If this component is deployed, the access control is enabled in the system, otherwise it is disabled.

Monitoring applications are not the only application logic that can be created in Appdaptivity. Actuation trigger and Alerts components enable the definition of automatic actuation and configurable notifications. For instance, a user can define the actuation with some devices (e.g., windows) when there is change in the environment (e.g, high temperature). Users can also receive notifications when the underlying infrastructure matches a configurable criteria. This application logic allows automatic behaviours and actuation without any user interaction. Thereby, the main goals of the IoT, sense and actuate over the physical world [2] have been included. Furthermore, in the cases where a specific logic is necessary, the Function component enables the inclusion of specific source code like a data mining algorithm in the JavaScript programming language. As can the rest of the components in Appdaptivity, the Function component can be added in run-time and the application logic will be adapted with it.

When behaviours become complex, programming can become a tedious task due to the number of PoCo components and necessary data flows to define them. For that reason, behaviours can be reused creating a subflow component. Subflow can be defined by selecting a group of PoCo components and selecting the option “create a subflow”. Once a subflow has been defined, a new PoCo component with the selected behaviours will be available in the system. Therefore, subflow components enable the programming reuse in addition to reducing the complexity of large flows and facilitating programming for non-expert users.

**Figure 6:**
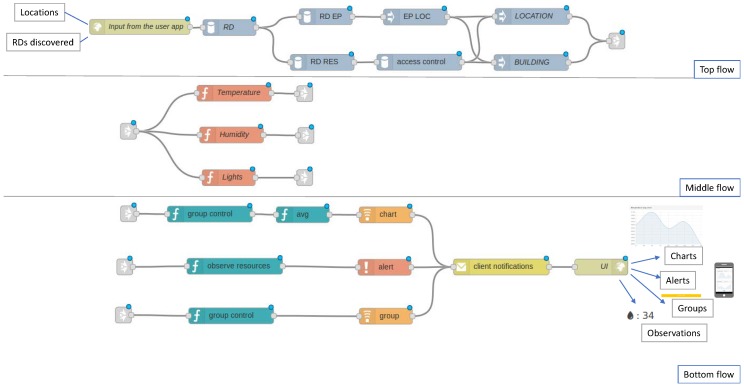
PoCo configuration with building and location flows, access control, and different behaviours.

Although the possible PoCo configurations are large, the configuration presented in Figure 6 covers most of the behaviours presented above. The top flow is responsible for the discovery of the IoT infrastructure, thanks to the observation of the end points (RD EP component) and resources (RD RES component). The next components allow it to get the end point locations and enable the location and building flows. Note that this flow can be grouped into a subflow to simplify the development. The middle block contains, from top to bottom, temperature, humidity and light filters respectively. Lastly, three different behaviours have been created in the bottom flow: an average chart of temperature, an alert of humidity, and a group of lights. As can be seen, Appdaptivity provides an intuitive way to program IoT applications with just a few components.

### 5.6. User Applications

The Appdaptivity architecture has been designed to be abstracted from user applications, in such a way that any application can be integrated with Appdaptivity using its communication means and understanding its data formats. Hence, in addition to user applications created from scratch such as smartphone, web or desktop, Appdaptivity can also be integrated into other platforms or applications through its API. As discussed, behaviours are defined in the PoCo and user applications display the information and data received to end users in the way that they have been configured. Taking into account the system goals, user applications should provide the mechanisms to access and interact with the underlying physical devices without the efforts of programming and configuring. This goal has been the key philosophy in the design of user applications, therefore configuration and behaviours are defined in the PoCo, whereas user applications are only responsible for displaying the behaviours defined, discovering RDs when the system requires it and establishing the users’ locations, which do not require any configuration. Consider, for example, a smartphone application launched as a user application in Appdaptivity, the only configuration required is installing the application if the application logic has previously been defined. Suppose a smart building, the administrator could create the application logic associated with the building infrastructure. Then, workers only have to install the application to start using the smart building functionalities.

**Figure 7:**
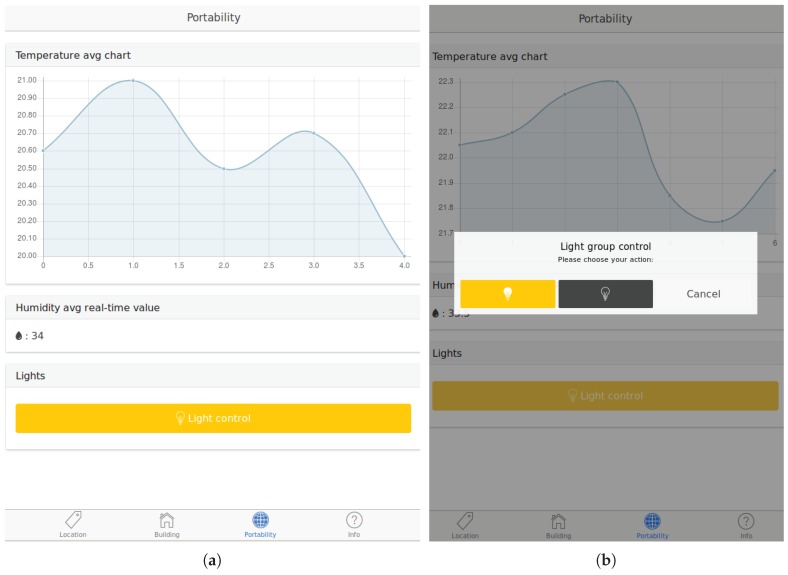
User application screenshots with some defined behaviours defined in the PoCo. (**a**) A chart, real-time values and a group of lights; (**b**) Pop-up window for interact with a light group.

Once the locations or the building flow have been established and started respectively, user applications start receiving the behaviours defined in the PoCo configuration, their corresponding data and the changes during the execution of the application. Figure 7 shows screenshots of a user application with an established location. Concretely, from top to bottom, Figure 7a displays an average temperature chart, a humidity average real-time value, and a group of light actuators for the interaction with. Figure 7b displays a pop-up window to manage a group of light actuators. The left button of the pop-up will turn on all lights belonging to the selected group once it has been pressed, whereas the center one will turn off all lights. User applications always contain up-to-date behaviours based on the state of the CoAP networks, and so in the case of a new or shutdown CoAP server deployment, user applications will be updated by the PoCo.

The RD discovering can also be done by user applications, especially in cloud deployments where the PoCo cannot discover RDs by itself. It is important then that the discovering service takes into account the network changes, as for example, the connection to another Wi-Fi network, since RDs could be deployed in such networks. Lastly, although optionally, user applications can interact directly with the resources received with a CoAP client, otherwise they can use the request service available in the PoCo.

## 6. Implementation

### 6.1. PoCo and Communications

PoCo has been implemented as an extension of Node-RED [21], an open source visual tool for data flow programming with a Web interface. Specifically, a set of nodes (PoCo components, communication and discovery) has been developed to be part of the Node-RED palette in order to address the requirements offered by the system. Each developed node has a specific function, such as the creation of a group of resources. The connection of these nodes comprises the behaviours that can be defined in Appdaptivity, as shown in Figure 6.

The communication between user applications and the PoCo has to be able to provide asynchronous communications since both components can send information asynchronously. Information can be dispatched from the PoCo to user applications continuously in groups or resources observations and sporadically when the CoAP network changes. User applications can also send asynchronous data with the RD discovered process and the location establishment. As a result, the communication channel has also to be bidirectional. Historically, applications that need bidirectional communication require different TCP connections, one for sending information and a new one for the reception. This entails the maintenance of multiple connections, mapping between each other and the abuse of HTTP polling for updates. WebSockets [39] was conceived as an alternative to HTTP polling providing two-way communication in a single TCP connection. In fact, WebSockets connections are done in the same ports as HTTP, nevertheless WebSockets and HTTP can only understand themselves in the handshake. Header and latency are usually smaller in WebSockets, thereby it is considered as a real-time communication channel. Moreover, WebSockets has also been integrated by default in most web browsers, the Web, and a large set of clients, so it opens the door to the integration of a large variety of clients and applications. Node-RED nodes have been developed in the PoCo to have a Websockets channel. The communication between user applications and the PoCo is encrypted leveraging the TLS/SSL support in WebSockets.

**Figure 8:**
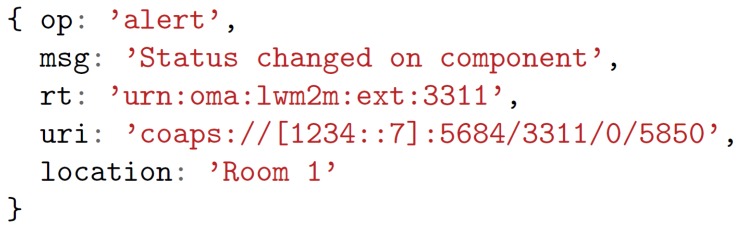
Alert message sent from the PoCo to an user application.

The data format is another key challenge in the development of applications, since bizarre and random data formats carry out into large efforts of development, code difficult to maintain and a door opened to multiple issues. We have therefore chosen JSON—an open-standard and de facto format in a large amount of applications and development frameworks—as message format. A semantic has been defined for the message definition. All messages exchanged from PoCo to user applications contain an operation type and most of them, a location, which indicates the operation has to be done in user applications and the location where the event has taken place, respectively. Optional values can also be included, for example, a message status, the resource type of a resource, the value of a request and information for rendering components. An example of an message exchanged between the PoCo and user applications is presented in Figure 8, specifically an alert message of the change of a light actuator. The message is an alert operation in the location Room 1, indicated in the fields op and location respectively. Other fields indicate the message status and the resource type and URI of the component. Messages are sent once new behaviours have been created for user applications, with the observed data from resources and operation results.

### 6.2. User Applications

Appdaptivity does not focus on a certain user application, but it paves the way to the integration of them into the system. To integrate external end users with Appdaptivity, an application should be defined with a WebSocket communication, which should understand the API defined. Moreover, the application should provide the capabilities to visualize the behaviours defined (e.g., real-time charts) and enable end users to interact with the system (e.g., group of actuators). Lastly, NFC reading capabilities will be required to change the location. To validate Appdaptivity we have created a smartphone user application. Smartphones have revolutionised the way in which we interact with each other and we use applications. Moreover, they are commonly used nowadays, and through them the release of a smartphone can be quickly extended amongst end users. To avoid the programming efforts in programming smartphone applications for different OSs, we chose a multi-platform framework for developing smartphone applications called Ionic [40]. The Ionic framework enables the application programming using web technologies such as HTML and JavaScript. It is based on AngularJS, a well-known framework for creating web applications. Once applications have been implemented in Ionic, they can be compiled for the target mobile OS such as Android and IOS. This reduces the development effort since only one source code is maintained.

The resulting smartphone application enables end users to switch locations using the NFC technology and render the behaviours as defined in the PoCo (e.g., charts, group of actuators and real-time values). Apart from the location flow, end users can also activate the building flow obtaining all the behaviours defined in the building where they are. Furthermore, the smartphone application provides support for network discovery required in Appdaptivity cloud deployments. Figure 7 shows some screenshots of the resulting application running some behaviours defined in the PoCo such as charts and group of lights once a location has been established. Although the application is intended to be installed in multiple mobile OSs, currently the application only offers support for the tested OS: Android.

In certain situations, user applications have to perform a network discovery, like, for example, the RD discovery in a cloud deployment. The RD can be discovered through IPv6 multicast using its defined interface. However, during the development of a smartphone app as a user application in Appdaptivity, we realised that IPv6 multicast was not working properly in some devices, especially in smartphones. In this case, we extended the Appdaptivity with multicast Domain Name System (mDNS), a zero-configuration service—it does not require manual configuration nor special servers—which shares similarities with DNS and resolves hostnames to IP addresses. mDNS is supported by Android and IOS OSs, and the open source implementation known as avahi, which has become the de facto standard implementation in Linux. In fact, the mDNS support in Appdaptivity uses the avahi implementation to publish a service on an established IP where clients can discover the RD in those cases where IPv6 multicast does not work properly. Smartphone-based user applications can make use of this service in order to reduce the issues using Ipv6 multicast as in our case.

### 6.3. CoAP Network

CoAP networks have been deployed using the CoAP++ framework [41], a CoAP framework which enables the definition of CoAP applications with extended capabilities such as support for group communication, ACLs, observe operation and conditional observe developed by the IDLab research group. The CoAP++ framework allows a run-time definition of ACLs that is used to manage which user can access which resources. This allows custom control, as for example, only enable the data access of the resource (GET method) or the interaction with (PUT method). The ACL can also support cipher suites for DTLS secured communication, including the certificate and public key and virtual resources in the CoAP server. An example of resource virtualisation is the location resource which typically is a virtualised resource that is hosted in the gateway, and not on the device itself. The communication process usually involves high-requirements in terms of communication, processing and memory footprint, which are not available in resource-constrained equipment, such as class 1 devices. The CoAP++ framework can be deployed in more powerful devices such as Raspberry Pi or BeagleBone and allows the use of pre-shared keys in order to avoid using public key operations in DTLS communications and reducing the requirements for performance-constrained environments [42].

The group communication was presented as an RFC in [43], nevertheless the CoAP group and the observe specification required to observe groups, have not been defined to work together [44]. This means that if a client needs an up-to-date representation of the resources belonging to a group, it has to continuously pull the group or observe the resources individually. In both cases, the client loses the benefits of the observe option and the benefits of the group, respectively. In the CoAP++ framework, the observe option and groups have been integrated and enable the possibility to apply different operations on groups of resources (e.g., average, minimum, maximum and list of values). Although group operations can be optional in some use cases, like, for example, actuating environments, and in other environments such as continuous monitoring of set of resources, the group communication in the CoAP++ framework provides an optimal solution. Any standard-based CoAP server and RD implementation could be used in Appdaptivity, however some optional functionalities not available in other implementations such as the group management and access control available in the CoAP++ framework could be required in some behaviours.

## 7. Results and Discussion

In this section an evaluation of Appdaptivity is presented. To evaluate the performance of Appdaptivity we have choosen different test scenarios. On the one hand, CoAP servers in the CoAP++ framework together with a lightweight user application without a graphical user interface (GUI) have been created to evaluate Appdaptivity with a large number of clients and CoAP devices. This scenario enables the inclusion of multiple user applications and devices to the CoAP networks without the need to use multiple hardware devices, thereby facilitating the realisation of performance tests. On the other hand, a real deployment using a smartphone user application and a CoAP network with physical devices including sensors and actuators has been deployed as use case of an emerging IoT area. Moreover, one use of Appdaptivity is illustrated in a smart home at the Ghent University. The performance tests have been done in a 4GB Xubuntu OS virtual machine running over a 8 GB Windows 7. Note that this virtualization could affect the evaluation done, especially in the real deployment. However, a virtualized environment was chosen for both the development and the evaluation processes in Appdaptivity. Due to its capababilites of replication, snapshot and isolation from the host OS.

### 7.1. Underlying IoT Infrastructure

**Figure 9:**
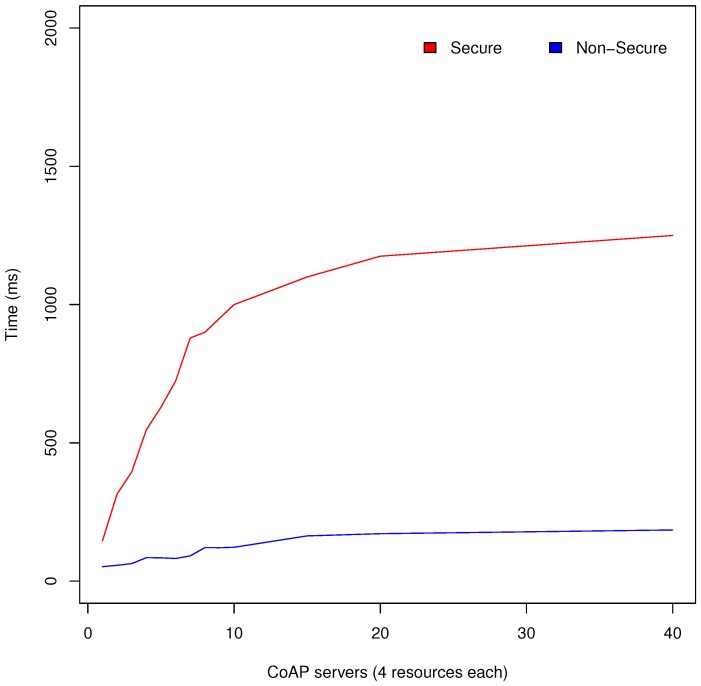
Location discovery response time with respect to the CoAP end points discovered.

In the tests, the total time until the resources and its locations from the CoAP network are obtained has been measured. This process includes the resources and end point collection from observation of the RD discovered, and the location collection from each end point obtained. Tests have been done with access control enabled (thereby with secure communication) and disabled in order to evaluate Appdaptivity in the different environments that it can be deployed in. Each CoAP server used in the CoAP network has 4 associated resources: temperature and humidity simulated sensors, a light simulated actuator and a location resource to obtain its physical location. Figure 9 shows average response times obtained from the performance tests done. As can be seen, the discovery time with access control enabled varies a little, whereas in the secure communication the discovery time increases more notably with respect to the number of CoAP servers.

Secure communications use DTLS which implies higher delays than UDP due to the necessary steps to secure the channel. However, Appdaptivity can manage these CoAP networks without large delays, and therefore it can be applied in large IoT deployments. In this test, it was also indented to evaluate the requirements of portable application development, thanks to the portability of IoT infrastructure (done thanks to the discovery) and personalised applications (secure configuration).

**Figure 10:**
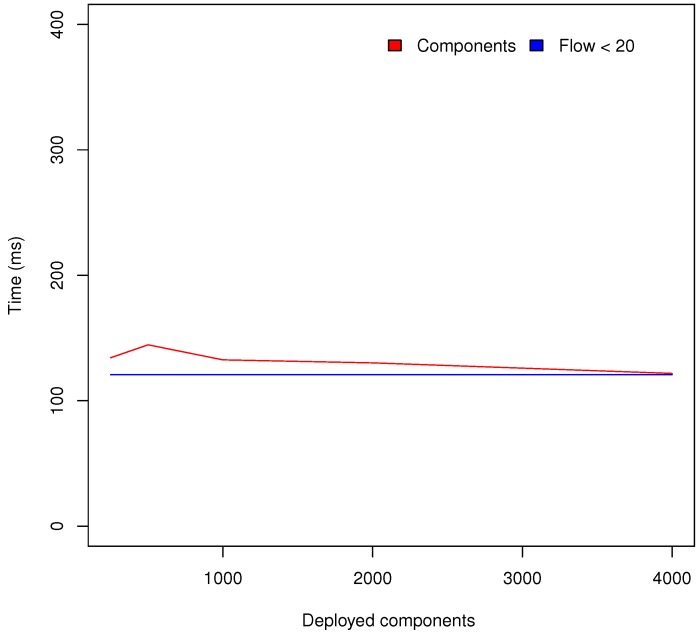
Location discovery response time with respect to the PoCo components deployed.

Deploying complex behaviours which involve a large number of PoCo components can reduce the scalability of the system. For that reason, the location discovery response time has once again been measured with 7 CoAP servers (4 resources each) and a different number of PoCo components. Figure 10 shows the results obtained with respect to a different number of PoCo components. In the same chart, the location discovery response time obtained in Figure 9, is also included which has fewer less than 20 PoCo components. As can be seen, the number of deployed PoCo components does not affect, to a great degree, to the scalability of Appdaptivity.

### 7.2. User Applications

**Figure 11:**
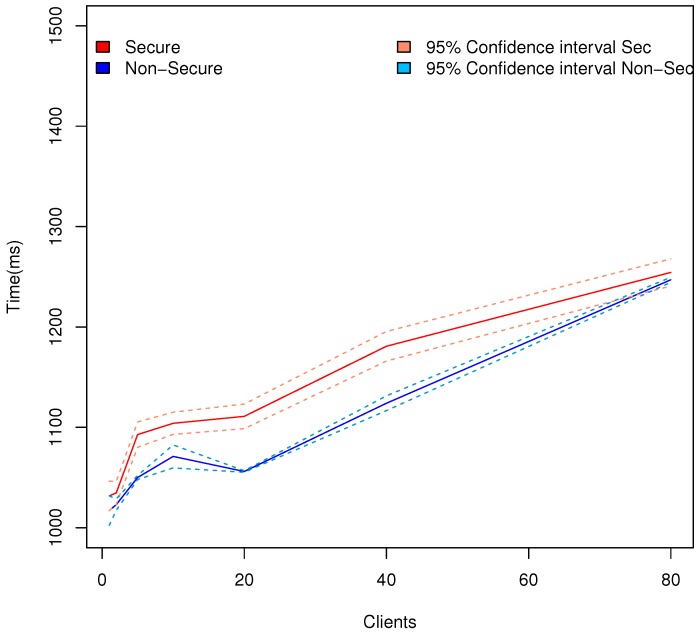
Response time with different numbers of user applications and the behaviour list of resources.

The number of connected users that Appdaptivity can handle can limit the possibilities of applying Appdaptivity in the IoT. In this test, we will evaluate the number of user applications that can interact concurrently with Appdaptivity. The tests have measured the response time since user applications send an RD discovered until the information of the behaviours defined (list of resources) is received with multiple user applications concurrently. Therefore, the location discovery time measured above is also included. The CoAP network comprises 8 CoAP end points along with their 4 corresponding resources as presented above. In the same line of the connection with the CoAP network, Appdaptivity also guarantees a secure and private communication with user applications through the fundamental protocol in the Internet transport security: TLS/SSL. In the comparison, the scenarios with a secure and non-secure communication channel have been included. Figure 11 displays the average response time, and the 95% confidence intervals in the aforementioned scenario. The variation between a secure and non-secure channel varies slightly until it remains stable with 80 user applications concurrently. Confidence intervals show a higher difference due to the use of secure communications. We have taken 80 users as the upper bound as an example of workers of a medium-size building. Therefore, in this case it is recommendable to activate the secure channel at all times. Appdaptivity can comfortably handle a large number of user applications. The worst case time may seem a bit high, however it only has to be done once and includes the communication response from all the clients, the discovery of the CoAP network and a CoAP request for each one of the 8 CoAP servers to get their locations.

### 7.3. Smart Cities: Portability of IoT Services in Different Districts

The design of personalised applications represents one the main potentials of Appdaptivity. The data flow programming approach and the range of behaviours that can be defined with the PoCo components enable the definition of applications in a large set of areas in the widely expanded IoT field. To demonstrate this potential, we define a real use case and translate it into Appdaptivity. The goal of this use case is to demonstrate how IoT applications can be created intuitively and agnosticly from the underlying IoT infrastructure with Appdaptivity. In addition, the portability of the solution and personalised behaviours using resource-constrained devices.

Consider a smart city with two districts. Both districts have different IoT services to allow the population to interact with, however the IoT services and networks can vary over the time. Therefore, to maintain the service continuity to the population without the need to install multiple applications or write specific and non-reusable code it is necessary to have a system that can cope with these challenges, and this is where Appdaptivity can be applied.

As mentioned above, the districts contain different IoT services. Let us suppose that in district D1, a light actuator is activated automatically when a presence is detected by a passive infrared sensor (PIR) or a illuminance sensor. The population could also query the luminosity measurements. On the other hand, in district D2, end users can control light actuators in a single way or individually, and see the average temperature in a chart.

**Figure 12:**
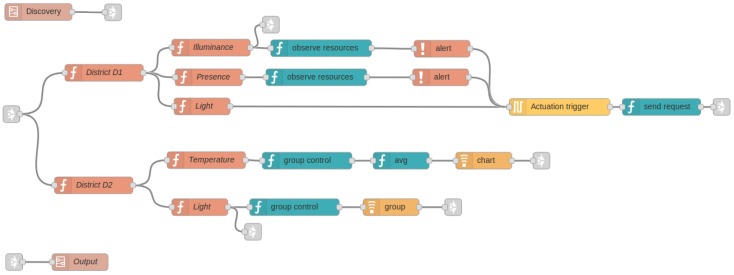
Smart-city use case translated into a Appdaptivity flow.

As the use case has different locations, the resulting data flow in the translation to Appdaptivity should differentiate the behaviours for each district. In the D1 district, the luminosity control is managed automatically through an Actuation trigger component which receives the list of lights to interact with and an alert once a presence has been detected or the illuminance is lower than a given limit (e.g., 30%). This component sends the list of lights to trigger the send request component which is responsible for making these requests. The population can query the illuminance sensors directly in their applications through a direct connection of the illuminance sensors to the user applications. In the D2 district, the population obtains a chart of average temperature. Lastly, the lights can be controlled directly, sending the light resources to the user applications, and controlled in a group-way with the creation of a group. Figure 12 displays the resulting Appdaptivity flow from the smart city use case presented. The mandatory components to enable the discovery of the underlying IoT and exchange information with the user applications have been grouped into two subflow components (Discovery and Output), so that the rest of components are PoCo components to define the use case behaviours. The development with just 20 components, shows the potential of Appdaptivity for the intuitive development of an IoT use case.

6LoWPAN has been choosen as the communication medium to deploy the smart city use case. 6LoWPAN enables the transmission of IPv6 packets over constrained networks, thereby providing an IPv6 address for each device that will be IP-accessible through the Internet. The CoAP network comprises four Zolertia RE-Mote (Zolertia RE-Mote platform: https://github.com/Zolertia/Resources/wiki/RE-Mote) devices, two for each district. The Zolertia RE-Mote is a hardware development platform which provides support for 2.4-GHz and 863–950-MHz IEEE 802.15.4 and ZigBee compliant radios with low consumption. Zolertia RE-Mote also provides support for the well-known open source OS for embedded devices, Contiki, and intrinsically for its 6LoWPAN implementation. Erbium, the Contiki REST engine and the CoAP implementation have been used to deploy the CoAP servers into Zolertia RE-Motes. As stated, Appdaptivity is decoupled from devices, thus Zolertia RE-Mote and Contiki have been used to validate the system but other devices and CoAP frameworks like Intel Edison and Californium can also be used with Appdaptivity. The fragmentation and reassembly mechanisms needed in 6LoWPAN to enable the communication with IPv6 networks are done by edge routers using RPL. We have chosen the Zolertia Orion router (Zolertia Orion Ethernet: https://github.com/Zolertia/Resources/wiki/Orion), an IPv4/IPv6 and 6LoWPAN routing device with Ethernet interface and 2.4 GHz and 863–950 MHz IEEE 802.15.4 radio support. The CETIC 6LBR (CETIC 6LBR: https://github.com/cetic/6lbr/wiki) border router solution has been installed into the Orion routers (one per district) to transparently enable users to communicate between IPv6 and 6LoWPAN networks. CETIC 6LBR also enables a Web UI to manage the router configuration and see its connected devices. A standard digital PIR sensor, the TSL2561 lux sensor with I2C connection, two DHT22 temperature and humidity digital sensors, and four standard digital LEDs were used as sensors and actuators in the use case. The experiments were performed in two of our laboratories (one per District), in the Research Building Ada Byron, University of Málaga. In each laboratory a NFC tag was place to change the location. The use case was active for one hour, with default max-age in the observe operation, thus generating approximately 300 CoAP messages.

**Figure 13:**
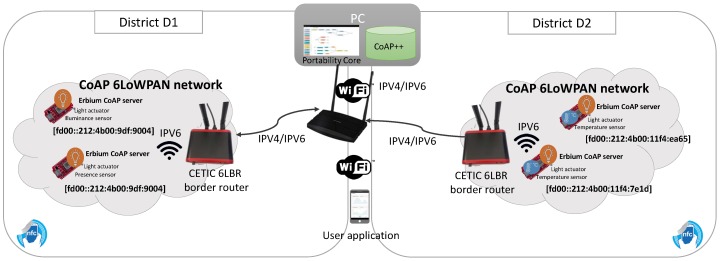
Smart-city use case deployment scenario.

The CoAP++ framework has been used to allocate the RD, where the Zolertia RE-Motes register their resources and end points and Appdaptivity observes it to get the status of the CoAP networks. Appdaptivity has been deployed in local model along with CoAP++ framework in a 4GB Xubuntu OS virtual machine running over a 8GB Windows 7. The border routers and the PC are connected to a switch router by Ethernet and Wi-Fi respectively. Finally, the user application has been installed on a Android smartphone which is also connected by Wi-Fi to the switch router and can change the district with the NFC tags located in each one. Figure 13 shows an overview of the smart city use case proposed. As mentioned before, each district has a border router with a 6LoWPAN network. End users can change the location with the NFC tags located in each district independently of the networks without interrupting the service continuity of Appdaptivity.

**Figure 14:**
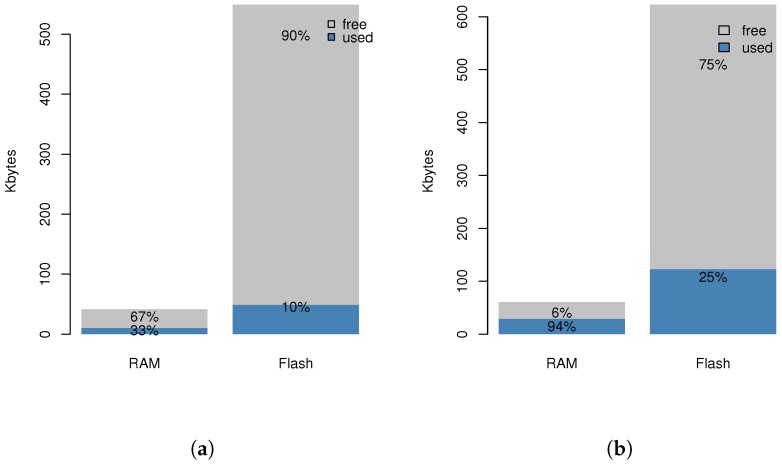
RAM and Flash memory usage in the Zolertia RE-Motes and the Zolertia Orion router. (**a**) Memory average usage in the sketches of the Zolertia RE-Motes; (**b**) Memory usage in the sketch of the Zolertia Orion Router.

Figure 14a,b display the RAM and Flash usage in the Zolertia RE-Motes and Orion routers respectively. This information has been obtained from the Contiki OS once the sketches have been uploaded to the devices. As can be seen, the Zolertia Re-Motes have 90% free space of Flash and 67% of RAM, allowing the creation of a large variety of new resources and reducing the power consumption thanks to the lightweight sketches. This test corroborates with the requirements of an embedded solution to reach resource-constrained devices, and the adoption of a current standard (CoAP). On the other hand, the Orion router needs more requirements: 75% of free space in Flash and 6% in RAM, however their sketches do not suffer many changes and they are usually connected without batteries. The power consumption evaluation resulted in an average 42 mA in the Zolertia motes which decreases the consumption more than two times of other hardware development platforms using 802.15.4 radios, as described in our previous work in [45]. The power consumption of the Zolertia Orion routers has not been measured since they require large power capabilities with the use of Ethernet and it is assumed that they are powered without batteries. Finally, the discovery process in Appdaptivity takes an average of 286.4 ms in a non-secure mode.

### 7.4. Appdaptivity in HomeLab

Appdaptivity has also been evaluated in a real scenario. In the video by one of this paper’s authors, Jen Rossey [46] you can find a demo tutorial of Appdaptivity for its use in HomeLab. HomeLab [47] is a standalone house at the Ghent University, designed as a test environment for IoT services and smart living. In the video you can see charts from power meter and temperature sensors in different rooms changed using the building flow. Moreover, it shows how the lights can be controlled with the inclusion of a new button component at runtime, showing the corresponding flows in the PoCo.

## 8. Differences between CoAP, the CoAP++ Framework and Appdaptivity

Consider that you want to create an application to manage and monitor some conditions in your work building or at home. You could deploy some CoAP servers together with some sensors and actuators. You could also get the same sensors and actuators for direct use from from lock-in vendors, but it is clear that this way only creates a vertical silo in the IoT. Then, you get resource-constrained devices to work with CoAP, such as Zolertia Re-Motes. In the next step, you would have to program the low level details for accessing these sensors and actuators in the Erbium framework. After that, you could interact with these sensors and actuators through CoAP, using for example the well-known CoAP web client Copper (Cu). However, you may also require other functionalities such as a HTTP-CoAP proxy, an RD, or being able to create groups of resources and conditional observations, and therefore decide to use the CoAP++ framework, installing it on a Raspberry Pi. Again, you can interact with the physical infrastructure with extended functionalities and the possibility to use any HTTP client to interact with the CoAP network, e.g., a web browser. Nevertheless, you would like to have a smartphone application to do the same tasks with your smartphone. You have decided to use the CoAP framework Californium and the Android platform to develop one. Now it is time to program your application logic. You can create automatic tasks that open the windows when the measurements of the CO2 sensors reach above a set level, a button to control all your lights and charts and alerts showing your location conditions such as temperature and humidity. Note that until this point Appdaptivity has not been used nor required.

Now, you have seen that the system works well with the use of open standards and you decide to control another room with new sensors and actuators. If you have programmed a specific functionality for the previous devices as is usually the case, you will have to program and re-compile the application to incorporate the new devices. Alternatively, your application may be able to automatically discover new CoAP servers together with the sensors and actuators of the new room, but normally you have to create an application with these new components. In these cases, you create device-coupled applications. Nevertheless, you could have created an application that automatically discovers the new devices in the CoAP networks and adapt the application logic as Appdaptivity does.

Let’s suppose you can control the illumination of your space. You can use the group communication of the CoAP++ framework to create a group for managing them. In the case that there are new lights or changes in them, Appdaptivity will update the corresponding behaviours, and also update their corresponding groups to ensure an up-to-date configuration. Furthermore, the rest of the behaviours that depend on these changes will also be updated, e.g., the trigger component that checks presence sensors to actuate over these lights. With Appdaptivity, if you control n components and you deploy a new CoAP server with the same component, you will have the opportunity to control n+1 components automatically. This can be extrapolated to other behaviours, e.g., if you are displaying statistics about conditions of some sensors, each change in these sensors will be detected by Appdaptivity and the associated configuration will be updated. Appdaptivity will also enable the portability of the IoT applications to new CoAP networks in the future. In the case of personalised applications the challenges are higher. In this case the smartphone application can be installed and will always count on an up-to-date representation of the CoAP networks, with the result that the applications are adapted to the context changes, can be personalised and portable. Therefore the aim of Appdaptivity is to complement CoAP and the IoT in the development of personalised, portable, adapted to changing context IoT applications.

## 9. Conclusions and Future Work

In this paper we have proposed Appdaptivity, a framework that enables the development of IoT applications abstracting application developers from the underlying physical infrastructure. The proposed solution reduces the gap between IoT application and the underlying physical infrastructure, which is actually highly coupled and carried to multiple vertical silos. The resulting applications are created intuitively, personalised, portable and automatically adapted to changing contexts without recompiling and programming device-coupled applications. The Appdaptivity programming model is based on data flow programming, where application developers can create IoT applications connecting components through a Web UI. Application developers only need to focus on the application logic, whereas the aspects of discovery and injecting a device’s information to applications are provided by Appdaptivity. The solution is intended for heterogeneous scenarios, enabling the portability of IoT applications, various multi-deployment options and the integration with external user applications. The underlying physical domain is supported through the Constrained Application Protocol (CoAP), an open standard to enable web services inside resource-constrained devices. Lastly, the final applications that will be used by end users, known as user applications, are not targeted to a specific application, but Appdaptivity enables the integration of multiple applications and platforms through its well-defined API and standards protocols.

The evaluation was performed in the different scenarios that can be part of Appdaptivity as secure and non-secure environments. The evaluation results confirm a good scalability of the system with respect to the user applications and the underlying physical infrastructure. Moreover, this work has also taken into account an evaluation in an IoT area. A smart city use case has been modeled using Appdaptivity and has been deployed with IoT devices and resource-constrained networks. 6LoWPAN networks have been deployed in the use case, allowing the devices to be IPv6-addressable through the Internet. As aligned with the requirements, an embedded CoAP open source implementation has been used in the evaluation for the IoT devices, but others that follow the standard or even other platforms can also be part of Appdaptivity. The evaluation also confirmed good results in terms of power consumption which, in turn, has an effect on the battery lifetime of the IoT devices. Finally, the use case shows us the potential of Appdaptivity to be applied in the wide range of areas which comprise the emerging IoT, as the deployment in HomeLab. The data flow programming model used in Appdaptivity allows multiple configurations, or behaviour such as those discovered in this approach, with only a few components and at the same time it enables the application development in a easy and non-technical way.

In future work, we will enable multi-user support in secure communications and automate the definition of virtual components in the CoAP++ framework. Changing location based on other identification technologies, would enable the creation of user applications for devices which do not support NFC. Autonomous composition of PoCo components is also planned. Moreover, we also intend to integrate RESTlet components. This can optimise the logic positioning between Appdativity and the underlying physical infrastructure enabling a distributed intelligence and the logic positioning in the component and at the right time.
